# Molecular Biology

**Published:** 1995

**Authors:** Alison M. Goate

**Affiliations:** Alison M. Goate, D.Phil., is an associate professor in the Department of Psychiatry, Washington University School of Medicine, St. Louis, Missouri

**Keywords:** AOD dependence, gene, research, laboratory method, molecular genetics, genome, genetic mapping, genetic linkage, environmental factors, etiology

## Abstract

Recent advances in molecular biology techniques permit scientists to identify genetic contributions to alcoholism. Two main types of technology are commonly used to identify genes that cause or predispose a person to a disease: positional cloning techniques and candidate gene techniques. Positional cloning techniques allow disease genes to be identified based solely on their location within the genome without prior knowledge of the gene’s function. Techniques for confirming the role of candidate genes rely on sufficient prior understanding of the disease process to implicate possible disease-related genes. Scientists use cloning techniques or the application of certain enzymes to reproduce a candidate gene in sufficient quantity for study. As the human genome project progresses and the gene map becomes increasingly complete, more and more disease genes will be identified through a combination of positional cloning and the candidate gene approach.

Alcoholism is a complex disease caused by a matrix of biological, psychological, and social factors. Although family, twin, and adoption studies have established a genetic contribution to alcoholism, the nature of this contribution is unknown. Researchers are using molecular biology techniques to identify and elucidate the mode of action of genes that predispose people to alcoholism.

## Genes and Disease

The genetic “blueprint” that determines the structure and composition of any living organism resides within the DNA molecule. DNA forms the backbone of each of the 23 pairs of chromosomes in the nucleus of every cell in the human body. A DNA molecule comprises long chains of chemical subunits called nucleotides.

Genes are specific sequences of nucleotides that provide the code for particular genetic traits. Each gene directs the synthesis of a different protein from chemical subunits called amino acids. The sequence of nucleotides in a gene determines the order of different amino acids in the finished protein and, hence, the nature of the protein.^1^

Some proteins form structural components of cells and tissues. Other proteins (e.g., enzymes) perform vital functions. A normal range of genetic variability contributes to diversity among the human population (e.g., racial characteristics and variation in eye color). Variation at a key site within a gene, however, may render the gene defective. Defective genes produce defective proteins; the resulting structural or functional abnormality forms the basis of genetic disease.

Chromosomes are inherited in pairs, one set of 23 from each parent. Consequently, each cell contains two copies of each gene. Diseases caused by a defect in a single gene are described as Mendelian, after the Austrian monk Gregor Mendel, who formulated many of the principles of inheritance in the mid-1800’s. Mendelian diseases fall into three classes: (1) dominant diseases, in which a single defective copy of one gene, inherited from either parent, is sufficient to cause the disease; (2) recessive diseases, in which both copies of the gene must be defective to cause the disease; and (3) sex-linked diseases, such as hemophilia, which are caused by a defective gene on the X (i.e., the female) chromosome.

Few diseases are inherited in straightforward Mendelian fashion; rather, many common diseases are complex in origin, caused by the effects of several genes interacting with the environment. Such diseases include heart disease, schizophrenia, manic-depressive disorder, and alcoholism. Researchers commonly use two approaches to identify genes that cause or predispose a person to a disease: positional cloning and the candidate gene approach.

## Positional Cloning Technology

Positional cloning comprises a group of techniques that allow disease genes to be identified based solely on their location within the subject’s total genetic material (i.e., genome)^2^ without any prior knowledge of the gene’s function. Genes so identified can then be copied (i.e., cloned) for study. This approach was used to identify the genes for both Huntington’s disease (Group 1993) and cystic fibrosis (Rommens et al. 1989). More recently, positional cloning has been used to identify chromosomal regions predisposing to diabetes (Davies et al. 1994). Positional cloning requires both a collection of families in which more than one member is affected by the disease under investigation and a comprehensive map of polymorphic (i.e., variable) genes to serve as reference points evenly spaced throughout the genome (see discussion of markers below). Researchers obtain DNA from certain cells in the subjects’ blood.

A common positional cloning technique uses restriction enzymes derived from bacteria. These enzymes cut DNA at sites marked by specific sequences of four to six nucleotide pairs (i.e., restriction sites), producing DNA fragments of different sizes.^3^ Because restriction sites are polymorphic, the size distribution of restriction fragments varies from person to person. This variation in fragment size is known as restriction fragment length polymorphism (RFLP).

**Figure 1:**
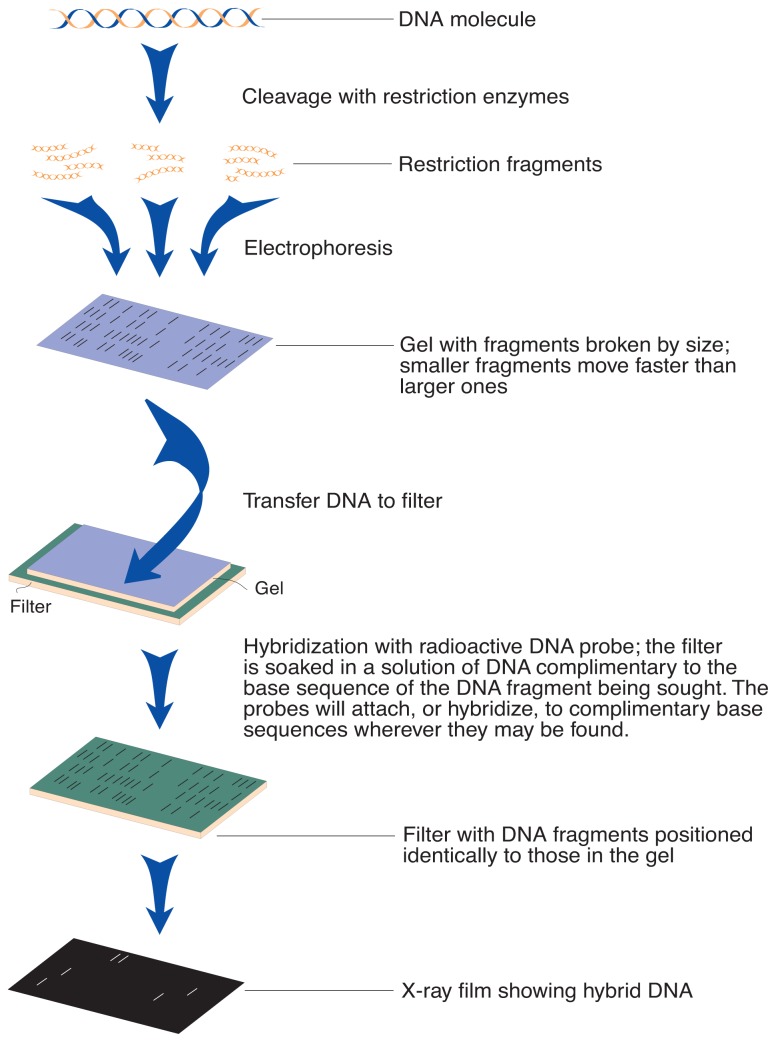
Separation of DNA fragments by electrophoresis and blotting. SOURCE: Adapted from Watson, J.D.; Hopkins, N.H.; Roberts, J.W.; Steitz, J.A.; and Weiner, A.M., eds. *Molecular Biology of the Gene*. 4th ed. Menlo Park, CA: Benjamin/Cummings Publishing Co., Inc., 1988. p. 609.

Restriction fragments can be sorted by size using a procedure called Southern blotting. First, the DNA is applied to one end of a gel and subjected to an electric current, a technique called eletrophoresis. The fragments migrate across the gel under the influence of the current, with smaller fragments moving faster. The DNA is then blotted from the gel onto a nylon membrane to facilitate identification of individual fragments. The membrane is soaked in a solution containing radioactively labeled DNA (i.e., a probe) designed to bind to a specific nucleotide sequence. The membrane is exposed to x-ray film to detect the polymorphic fragments, which appear as different banding patterns on the film (figure 1). If the location of a given band is consistently associated (i.e., linked) with a disease, then this pattern can be used as a marker for that disease.

Another method for detecting polymorphisms is the polymerase chain reaction (PCR). The enzyme DNA polymerase can copy a section of DNA if a short piece of DNA called a primer has been bound to each end of the section. The primer directs the DNA polymerase to produce multiple copies of the section in a chain reaction called amplification. The DNA section, generally amplified more than a million-fold, can be fragmented by a restriction enzyme and separated by electrophoresis. The creation of multiple DNA copies by amplification eliminates the need for a sensitive detection system, such as a radioactive probe.

The main drawback with RFLP’s is that most restriction sites exhibit only two possible variations—the site is either present or it is not. Consequently, only a small number of RFLP’s are genetically informative, permitting an unambiguous determination of which alleles (i.e., variants of a given gene) in the offspring are inherited from each parent.

Today the use of RFLP’s has been replaced largely by processes using microsatellite repeat markers. These markers are usually repeating sequences of two, three, or four nucleotide pairs randomly spaced throughout the genome. They are highly polymorphic, having as many as 10 alleles, and can be detected by PCR (Weber and May 1989). The polymorphism reflects variation in the length of the marker and can be detected using PCR amplification, gel separation, and radioactive or fluorescent primers. Radioactively labeled products are detected by exposure to x-ray film (Weber and May 1989), and fluorescently labeled products are detected by laser technology (Davies et al. 1994). Unlabeled PCR products also can be detected by blotting the gel onto a nylon membrane and then probing the filter with a radiolabeled PCR primer (Weissenbach et al. 1992). By using several fluorescent dyes, up to 15 different polymorphisms can be assessed simultaneously.

Using statistical methods, researchers can determine whether a marker and a disease gene are close enough together on a chromosome to be inherited as a unit (a condition referred to as linkage). Polymorphic microsatellite markers are usually selected for study such that 300 to 400 markers can cover the entire genome. Maps containing several thousand such markers are now available (Weissenbach et al. 1992). If markers are spaced too widely along the genome, there might not be any marker close enough to the disease gene to exhibit linkage. When a linkage is detected (generally by statistical analysis of inheritance patterns), additional microsatellite polymorphisms from the same region of the chromosome are tested to confirm co-inheritance with the disease and to determine the precise location of the disease gene with respect to the polymorphic markers. A variety of techniques can then be used to identify genes within the region where the putative disease gene (i.e., candidate gene) is located (Group 1993). Each gene will then be tested as a candidate gene, as described in the following section. If a highly likely candidate gene is already known to occur within the linked region, it can be tested immediately after linkage has been detected.

## Candidate Gene Approach Technology

Techniques for confirming the role of candidate genes rely on sufficient prior understanding of the disease process to implicate possible disease-related genes. Formerly, scientists used cloning techniques to reproduce a candidate gene in sufficient quantity for study. The cloning process generally involved propagating fragmented human DNA within a bacterium. The gene fragment of interest would then be isolated and its nucleotide sequence determined.

Currently, if the nucleotide sequence of a gene is already known from another species, the gene can often be purified from a batch of total human DNA by selective amplification using DNA primers capable of binding to the specific DNA sequence of the gene.^4^ The technique of amplification has revolutionized molecular biology by speeding up many processes and by reducing the need to clone genes.

### Association Techniques

The association approach has been used to identify gene variants that might predispose a person to a disease. Association tests whether a particular polymorphism is observed more frequently in a group of test subjects, who have the disease, than in a group of control subjects, who do not have the disease.

Polymorphisms can be detected using a variety of techniques. For example, the DNA sequence of interest can be amplified by PCR and treated with a restriction enzyme to produce RFLP’s. If the polymorphism does not create or destroy a restriction enzyme site, then several alternative approaches can be used. One such method is single-stranded conformation analysis, which compares a DNA sequence from a test subject with the corresponding sequence from a control subject based on the strands’ migration through a gel. Differences in the migration of test DNA and the control sequence provide evidence that the nucleotide sequence of the test DNA differs from that of the control DNA.

Two common problems exist with the association approach. First, a spurious result may occur if the control subjects are not properly matched to the disease subjects by ethnic and other factors that reflect genetic composition. Second, association alone does not rule out the possibility that the DNA variant is merely located very close to the disease gene on the chromosome. Biochemical experiments are necessary to demonstrate that a DNA sequence variant actually contributes to the development of the disease.

An association approach recently has been used to identify a polymorphism that may increase a person’s risk of developing Alzheimer’s disease; the presence of multiple copies of the disease allele increases the risk of developing the disease (i.e., dosage effect) (Corder et al. 1993). Another allele of the same gene, however, appears to decrease the risk for the disease (Corder et al. 1994). Together, these findings support the idea that the polymorphism is associated with the disease, although the precise biological effect of the sequence variants is not known.

A similar approach was used to study alcoholism. One allele of a polymorphism within the D_2_ dopamine receptor gene^5^ was reportedly associated with an increased risk of developing alcoholism (Blum et al. 1990). Further research, however, suggests that this result may be spurious (Gelernter et al. 1993).

### Linkage

An alternative approach is to study the inheritance pattern of a polymorphism within a candidate gene in families in which more than one member is affected by the disease under investigation. If the candidate gene is located near the disease gene on the same chromosome (or if it is the disease gene), then one allele of the polymorphism will be co-inherited with the disease more often than chance alone would predict within a family.^6^ In these circumstances, the polymorphism and the disease gene are said to be linked (Hsiao et al. 1989; Goate et al. 1991).

### Screening for Polymorphisms

If evidence from genetic linkage studies in families or genetic association studies in populations suggests involvement of a candidate gene in causing a disease, then the next step is to sequence the DNA of the gene to determine the variation in the nucleotide sequence that produces a change in the amino acid sequence of the protein (Hsiao et al. 1989; Goate et al. 1991). The most comprehensive way to screen a candidate gene is to sequence the DNA from affected and unaffected subjects. However, if the gene is large, this process may involve considerable work. Techniques like single-stranded conformation analysis can analyze genes in small pieces (i.e., a few hundred nucleotide pairs). Only a small piece of the DNA then need be sequenced to confirm the presence of a DNA sequence variant.

Although these techniques are rapid and enable screening of large numbers of samples, their main drawback is that they may not detect all DNA sequence variants. In addition, if a sequence variant is identified, it could represent either a normal DNA polymorphism or a defective (i.e., disease) allele.

Variation in the DNA sequence between individuals is extremely common but rarely leads to disease: Many stretches of DNA along the chromosome do not code for anything, and the DNA code itself is highly redundant. With four different nucleotides available, 64 possible 3-nucleotide combinations can code for the 20 amino acids found in proteins. Therefore, some polymorphism can occur in a coding sequence without altering the protein.

In a dominant disease, the defective allele will be present in one or both copies of the gene in affected subjects but not at all in normal subjects. In a recessive disease, the defective allele will be present in both copies of the gene in an affected subject and in one copy of the gene in carriers. In complex diseases, the alleles predisposing to disease also may be present in unaffected subjects, making it more difficult to determine whether a particular variant influences risk for disease. However, any sequence variant that influences risk for the disease would still be expected to show association with the disease.

## Conclusion

With the increasing number of polymorphic markers identified on the genetic map, positional cloning has become an extremely powerful tool, permitting the linkage and identification of a large number of disease genes. As the human genome project progresses and the gene map becomes as dense as the microsatellite polymorphism map, scientists will identify more disease genes through a combination of positional cloning and the candidate gene approach.

## Footnotes

1 There are four types of nucleotides; individual amino acids are encoded by specific sequences of three nucleotides.

2 For technical terms not defined in the text, see central glossary, pp. 182–183.

3 Because DNA is double-stranded, the distance along the chromosome is measured in nucleotide pairs. Only one strand contains the genetic code per se. The double-strandedness of the DNA molecule is a separate phenomenon from the existence of paired genes and chromosomes.

4 This technique relies on the substantial similarity of many genes across species.

5 This gene directs the synthesis of a brain protein thought to be involved in certain aspects of the addiction process.

6 The specific allele co-inherited with the disease may differ between families.
